# Gibberellin Overproduction Promotes Sucrose Synthase Expression and Secondary Cell Wall Deposition in Cotton Fibers

**DOI:** 10.1371/journal.pone.0096537

**Published:** 2014-05-09

**Authors:** Wen-Qin Bai, Yue-Hua Xiao, Juan Zhao, Shui-Qing Song, Lin Hu, Jian-Yan Zeng, Xian-Bi Li, Lei Hou, Ming Luo, De-Mou Li, Yan Pei

**Affiliations:** Biotechnology Research Center, Southwest University, Beibei, Chongqing, China; East Carolina University, United States of America

## Abstract

Bioactive gibberellins (GAs) comprise an important class of natural plant growth regulators and play essential roles in cotton fiber development. To date, the molecular base of GAs' functions in fiber development is largely unclear. To address this question, the endogenous bioactive GA levels in cotton developing fibers were elevated by specifically up-regulating GA 20-oxidase and suppressing GA 2-oxidase via transgenic methods. Higher GA levels in transgenic cotton fibers significantly increased micronaire values, 1000-fiber weight, cell wall thickness and cellulose contents of mature fibers. Quantitative RT-PCR and biochemical analysis revealed that the transcription of sucrose synthase gene *GhSusA1* and sucrose synthase activities were significantly enhanced in GA overproducing transgenic fibers, compared to the wild-type cotton. In addition, exogenous application of bioactive GA could promote *GhSusA1* expression in cultured fibers, as well as in cotton hypocotyls. Our results suggested that bioactive GAs promoted secondary cell wall deposition in cotton fibers by enhancing sucrose synthase expression.

## Introduction

Cotton is the leading natural fiber for textile industry worldwide. Biologically, cotton fibers are extremely elongated single-celled trichomes originating from outermost layer of ovule epidermis [1]–[4]. The development of cotton fiber may be divided into 4 stages, i.e. initiation, elongation, secondary cell wall deposition and maturation. Secondary cell wall deposition starts at around 14–17 days post anthesis (dpa) and lasts for over 30d [1], [3], [5]. In this stage, cellulose is intensely deposited to form a thick secondary cell wall. At maturation, cotton fiber consists primarily of secondary cell wall and over 90% dry weight of fiber may exist as cellulose. Therefore, carbon partitioning to cellulose biosynthesis is a key determinant of fiber weight and qualities, such as fiber strength and fineness [1], [3], [5]–[8]. Many efforts have been taken to reveal the role of genes involved in the regulation of secondary cell wall deposition and to manipulate them by genetically modification for improvement of cotton yield and quality [1], [6], [7], [9], [10]. Recently, Jiang and coworkers showed that over-expressing a cotton sucrose synthase gene, *GhsusA1*, enhanced thickening of secondary cell wall and fiber qualities, suggesting an important role of sucrose synthase in controlling carbon partitioning to cellulose biosynthesis in cotton fibers [6].

Gibberellins (GA) are a class of important plant hormones involved in many physiological and developmental processes, including seed germination, cell elongation, photomorphogenesis, flowering and seed development [11]. In the last two decades, the molecular base of GA biosynthesis pathway and its regulation have been largely clarified in model plants [12]–[14]. Endogenous bioactive GA contents are regulated mainly through three 2-oxoglutarate-dependent dioxygenases, i.e. GA 20-oxidase (GA20ox), GA 3-oxidase (GA3ox) and GA 2-oxidase (GA2ox) [13], [14]. GA20ox and GA3ox catalyze the last two steps to synthesize bioactive GAs, while GA2ox convert bioactive GAs and their precursors to inactive 2-hydroxylated forms. A wealth of evidence demonstrated that both up-regulating GA20ox and suppressing GA2ox could significantly increase endogenous bioactive GA levels and lead to GA overproduction phenotypes in plants [15]–[20].

Physiological and molecular studies have revealed that GAs played important roles in fiber development. Exogenous application of GAs *in vitro* and *in planta* promoted fiber initiation and elongation [21], [22]. Recently, we showed that over-expression of *GhGA20ox1* in cotton significantly increased bioactive GA level and promote fiber initiation and elongation at early stage [17]. However, global up-regulation of GAs leaded to overgrowth of plant and somewhat negatively affected fiber development, especially at the late developmental stage. Instead, it is reasonable to elucidate GA roles in fiber development by tissue specific regulation of GA levels in developing fibers. To this end, we elevated the endogenous active GA levels in cotton fibers by tissue-specific up-regulation of GA20ox gene and down-regulation of GA2ox gene. We found that enhancement of GA production in fibers promoted sucrose synthase expression and secondary cell wall deposition. Our results implied that GAs might enhance carbon partitioning to cellulose and secondary cell wall synthesis via up-regulating sucrose synthase expression in cotton fibers.

## Materials and Methods

### Plant material and growth condition

Upland cotton (*Gossypium hirsutum* L. *cv.* Jimian No. 14) was used for cotton transformation and GA treatment. Cotton seedlings were grown in a greenhouse with a 16h/8h (light/dark) schedule and temperature kept at 26–30°C. Fibers and ovules were collected from field-grown cotton plants at growing season in Chongqing, China.

For GA_3_ treatment of hypocotyls, 4-day-old seedlings were immersed in distilled water (pH6.0) or GA_3_ solutions of various concentrations (0.05 mM, 0.1 mM and 0.5 mM, pH6.0) for 48 h. Then the hypocotyls were measured and collected for expression analyses and cellulose determination.

For GA treatment of *in vitro* fibers, cotton ovules were cultured as described by Beasley [23]. Cotton bolls were harvested at 0-dpa and surface-sterilized in 75% (v/v) ethanol for 1 min, rinsed in sterile water, then soaked in 0.1% w/v HgCl_2_ solution for 12 min for sterilization, followed by rinsing with sterile water for six times. Ovules were separated, floated on BT media containing 5 µM IAA and GA_3_ of various concentrations (0.5, 2.5, 10 and 25 µM), and then incubated in darkness at 32°C for 20d. Fibers were striped from ovules and used for RNA extraction.

### Vector construction and plant transformation

To construct specific expression vector of cotton GA20ox (*SCFP::GhGA20ox1*), the CaMV 35S promoter in an over-expression vector (*35S::GhGA20ox1*) [17] was replaced with SCFP promoter[24]. The BAN promoter was amplified from Arabidopsis with a forward primer (5′-TCTAGATAACAGAACCTTAC TGTAACACTATT-3′) and a reverse primer (5′-ACTAGTGATTGTACTTTTGAAATTACAGAG AT-3′) and cloned into TA cloning vector pMD19-T (TaKaRa, Dalian, China). After sequencing, the BAN promoter was digested from the cloning vector by *Hin*dIII and *Bam*HI and inserted into a basic expression vector p5 vector [25] digested with the same enzymes to generate the vector p5-BAN. An intron-containing hairpin RNA construct of cotton GA20ox gene (*GhGA2ox2RNAi*) was amplified from cotton genomic DNA as previously described [26]. The 25-µl PCR mixture included 100 ng cotton genomic DNA, 10×Ex Taq buffer (TaKaRa), 200 µM each dNTPs, 2 mM MgCl_2_, 400 nM flanking primer (5′-GTATTGGTCTGGTGGGACTG-3′), 40 nM bridge primer (5′-CAAGTATCTCACATGCC AAGACCCGAATTCTCCTTG-3′), 1.5U Ex Taq DNA polymerase. The PCR thermo cycling parameters were as follows: 94°C for 5 min, followed by 35 cycles of 94°C for 30 s, 56°C for 30 s and 72°C for 30 s, and a final extension of 10 min at 72°C. The *GhGA2ox2RNAi* fragment was cloned and sequenced, then inserted into p5-BAN using *Bam*HI and *Kpn*I. Transgenic plants were generated using *Agrobacterium*-mediated transformation as described [25]. Based on expression analysis of target genes in transgenic cottons, two homologous transgenic lines were obtained by self-crossing, and their performances were documented at T3 and T4 generations in comparison with untransformed acceptor line (Jimian No. 14) grown in parallel in the field.

### RNA extraction and qRT-PCR analyses

Total RNA was extracted from roots, hypocotyls, leaves, petals, anthers, ovules and fibers using a rapid plant RNA extraction kit (Aidlab, Beijing, China). The single-stranded cDNAs were synthesized from total RNA using a cDNA synthesis kit (TaKaRa, Dalian, China). The gene-specific primers used for real-time PCR amplification were list in table S1. Cotton *histon3* gene (AF024716) was amplified as internal standard [27]. Real-time PCRs were performed on a CFX96 real-time PCR detection system with SYBR Green supermix (Bio-Rad, CA, USA). The thermocycling parameters were as follows: 95°C for 2 min, followed by 40 cycles of 95°C for 30 s, 56°C for 30 s and 72°C for 30 s, followed by a standard melting curve to monitor the specificity of PCR products. The reactions were duplicated for 3 times and data were analyzed using the software Bio-Rad CFX Manager 2.0 provided by the manufacturer.

### Determination of endogenous GA contents

Cotton fibers (200 mg FW) were ground to fine powder in liquid N_2_, extracted overnight in 5 ml 80% methanol at −20°C and deuterium-labeled [17, 17-^2^H_2_] GA_1_ and [17, 17-^2^H_2_] GA_4_ (each 10 ng) from Prof. L. Mander (Australian National University) were added as internal standards. After centrifugation, supernatants were collected, dried in a rotavapor (BUCHI, Switzerland) at 40°C, and re-suspended in 3 ml 10% methanol. The extracts were applied on Oasis HLB extraction cartridges (60 mg, Waters) pretreated with 3 ml methanol and 3 ml water. After washing with 1 ml 10% methanol, GAs were eluted with 1 ml 90% methanol. The eluates were evaporated to dry, dissolved in 100 µL 10% methanol, and subjected to LC-MS assay. The procedures for LC-MS quantification of GAs were described previously [17].

### Measurement of fiber quality

Mature fibers were harvested from the field-grown cotton in the same period (Aug. 20 to Sep. 10). After ginning, fibers were mixed well and 6 repeats of 10 g fibers were randomly sampled for each material. Fiber sample were tested independently for fiber quality traits (fiber length, fiber strength, micronarie value) using at a HVI system (HFT 9000, Uster Technologies, Swiss) in Cotton Fiber Quality Inspection and Testing Center, Ministry of Agriculture of China (Anyang, Henan, China).

### Microscopic measurement of fiber cell wall thickness

Statistical analysis of cell wall thickness was performed according to Wang *et al*. [7]. After fixing in FAA (37% formaldehyde: acetic acid: ethanol: water, 10∶5∶50∶35) at 25°C for 12 h, mature cotton fibers were dehydrated gradually in alcohol and tert-butyl alcohol series,and then infiltrated in tert-butyl alcohol/paraffin at 65°C and embedded in paraffin. The samples were sliced into 7-µm sections. The slices were mounted, stained with the Fast Green dye and photographed by a BX41TF light microscope (Olympus, Japan). Image-pro Plus program (Olympus) was employed to measure the thickness of cell wall and 1000 sections were measured for each sample.

### Determination of fiber weight

Fibers on seeds were combed straight and striped manually. Approximately 1.5 mg fibers were randomly bundled and weighed precisely (W1). The fiber number of each fiber bundle (N) was counted as described[28]. The weight of 1000 fibers (W2) was calculated from the following equation: W2 = 1000W1/N. For each material, the average 1000-fiber weight was calculated on the basis of 60 fiber bundles from different seeds.

### Sucrose synthase activity assays

The sucrose synthase was extracted according to Jiang et al. [6]. Fresh fibers (around 0.5 g) were ground to fine powder in liquid N_2_. The grinding continued for 5 min in cold extraction buffer (25 mM Hepes–KOH (pH 7.3), 5 mM EDTA, 1 mM DTT, 0.1% soluble PVP, 20 mM β-mercaptoethanol, 1 mM PMSF and 0.01 mM leupeptin). The homogenate was separated by centrifugation (10000 g, 5 min, 4°C) and the supernatant was used as the crude extracts for assays. Protein concentrations were determined via Bradford method [29]and sucrose sythase activities were assayed as previously described [30], [31].

### Analyses of soluble sugar contents

Fresh fibers (around 50 mg) were separated from developing bolls and ground to fine powder in liquid N2. The powder was extracted in 2 ml 80% (v/v) ethanol at 80°C?for 15 min. After centrifugation (3000 g, 10 min), supernatants were collected. The pellets were further extracted twice, and supernatants were combined and used for soluble sugar assays. The contents of glucose, fructose and sucrose were measured at 340 nm with a Synergy HT microplate reader (BioTek, Vermont, WS) as described [32].

### Determination of cellulose content

Cellulose contents were determined according to Wang *et al*. [7]. Around 0.1 g fiber samples were extracted in 10 ml boiling acetic/nitric reagent (80% acetic/nitric, 10∶1) for 1 h, then rinsed three times with distilled water and once with ethanol. Residuals were dried at 105°C for 2 h. The weight ratio of residual to initial samples was regarded as cellulose content. Hypocotyls were excised from seeding (10 hypocotyls per sample) and ground to fine powder in liquid N_2_. Samples were dried at 105°C for 2 h and extracted in 10 ml boiling acetic/nitric reagent (80% acetic/nitric, 10∶1) for 1 h.Determination of cellulose content of per hypocotyls was carried out as described [33].

### Statistical analyses

Performances of transgenic materials were compared to wild-type control and statistical significance of divergence between averages was determined by t test. All statistical calculations were performed using Microsoft Excel.

## Results

### Enhancement of GA production in cotton fiber

**Figure 1:**
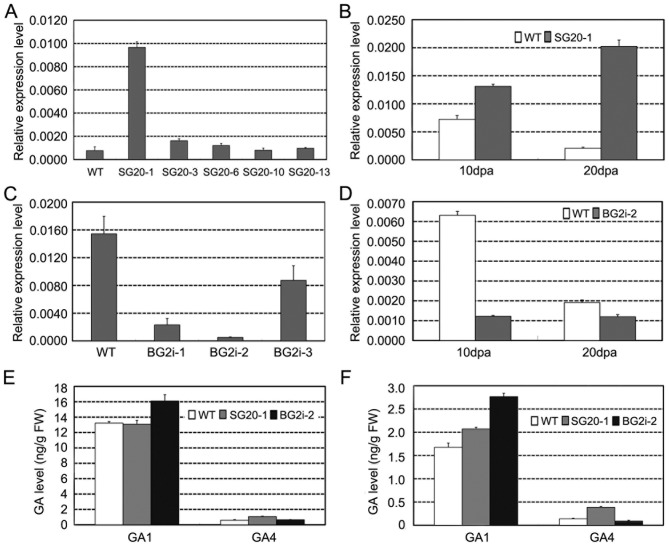
Up-regulation of *GhGA20ox1* or suppression of *GhGA2ox* in cotton fibers. (A) Quantitative RT-PCR analysis of *GhGA20ox1* in 5-dpa fibers of T_0_ *SCPF::GhGA20ox1* lines (SG20) and the wild-type cotton (WT). (B) Quantitative RT-PCR analysis of *GhGA20ox1* in 10-dpa and 20-dpa fibers of SG20-1 (T_3_generation) and WT. (C) Quantitative RT-PCR analysis of *GhGA2ox2* in 5-dpa fibers of T_0_ *BAN::GhGA2oxRNAi* lines (BG2i) and WT. (D) Quantitative RT-PCR analysis of *GhGA2ox2* in 10-dpa and 20-dpa fibers of BG2i-2 (T_3_generation) and WT. (E) Endogenous GA_1_ and GA_4_ content in 8-dpa fibers of SG20-1, BG2i-2 and WT. (F) Endogenous GA_1_ and GA_4_ contents in 20-dpa fibers of SG20-1, BG2i-2 and WT.

We used two strategies to tissue-specially enhance GA production in fibers, i.e. to promote GA biosynthesis by up-regulation GA 20-oxidase (GA20ox) and suppressing GA deactivation by down-regulation of GA2-oxidase (GA2ox). To this end, we used a fiber-specific promoter (SCFP) [24] and a seed coat- and fiber-specific promoter BAN [28], [34] to direct the expression of *GA20ox* (*GhGA20ox1*) [17], and *GA2ox*, respectively. Among *SCFP::GhGA20ox1* transgenic cottons (SG20), SG20-1 showed dramatically increase in *GhGA20ox1* expression level in developing fibers (Figure 1A and 1B).

We compared the expression pattern of six cotton GA 2-oxidase genes (*GhGA2ox1-6*, Figure S1∼3). Among them, *GhGA2ox2* showed predominant expression in fibers. Thus we selected *GhGA2ox2* as RNAi target to suppress GA deactivation in fibers, and generated *GhGA2oxRNAi* transgenic cottons. Real-time RT-PCR revealed that the expression level of *GhGA2ox2* was reduced in *BAN::GhGA2oxRNAi* (BG2i) transgenic cottons (Figure 1C), in which transformant BG2i-2 showed most significant suppression of the target gene in fibers (Figure 1C and 1D).

To detect the effect of *GhGA20ox1* up-regulation and *GhGA2ox2* down-regulation on GA homeostasis in fibers, we determined the contents of endogenous bioactive GAs (GA_1_ and GA_4_) in 8- and- 20 dpa fibers of SG20-1 and BG2i-2 by LC-MS (Figure 1E and 1F). Compared to the wild-type control, GA_4_ contents in the 8- and 20-dpa fibers of SG20-1 fibers increased 83.1% and 178.6%, respectively, while GA_1_ content was moderately increased (24.0%) in the 20-dpa fibers(Figure 1E and 1F). In BG2i-2 fibers, GA_1_ level was 21.6% and 65.9% higher than the control at 8- and 20-dpa respectively, whereas GA_4_ contents remained almost unchanged compared to the control (Figure 1E and 1F).

### Effects of elevated GA levels on secondary cell wall thickening of cotton fiber

**Figure 2:**
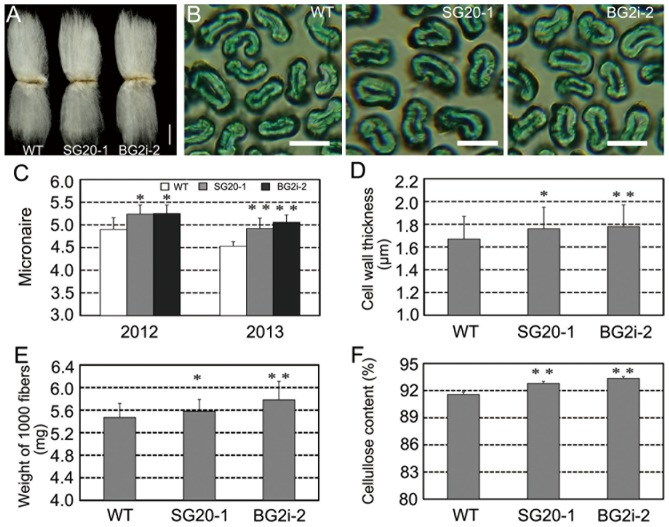
Effects of GA overproduction on secondary cell wall deposition in cotton fibers. (A) Cotton fibers on seeds; bar = 1 cm. (B) Cross-section of mature cotton fibers; Bar = 25 µm. (C) Micronaire values of cotton fibers in 2012 and 2013, n = 6. (D) Cell wall thickness of mature cotton fibers. (E) Weight of 1000 cotton fibers, n = 60. (F)Cellulose contents of mature cotton fibers, n = 6Asterisks (_*_) and double asterisks (_**_) represent significant differences(*t* test) at p = 0.05 and p = 0.01 compared with the wild type, respectively.

To clarify the effect of elevated GA levels on fiber development and fiber quality, we compared the agronomy performances of transgenic lines SG20-1 and BG2i-2 with the wild-type control in consecutive two-year field trails. No significant change in plant growth, yield traits and fiber length and strength (Figure 2A and S4; Table S2 and S3) was found between the transgenic lines and the wild type, except micronaire value. The micronaire values of SG20-1 and BG2i-2 fibers were significantly higher than that of the wild type (Figure 2C). Micronaire value is a composite measure of fiber maturity and fineness. To clarify whether the fiber fineness was increased in transgenic cotton, we measured the weight per 1000 fibers. The weights of SG20-1 and BG2i-2 mature fibers significantly enhanced in comparison with the control (2.0% and 5.7%, respectively; Figure 2E). Microscopic observation further confirmed that the cell walls of SG20-1 and BG2i-2 mature fibers were thicker (5.4% and 6.6%, respectively) than the wild-type control (Figure 2B and D). Considered that most of cell walls of mature cotton fibers consisted of secondary cell wall [2], it was reasonable that the fineness increase in SG20-1 and BG2i-2 fibers might be mainly attributed to promotion of secondary cell wall deposition. To prove this hypothesis, we determined the cellulose contents in fibers, and found that the contents of SG20-1 and BG2i-2 fibers were significantly higher than the control (Figure 2F). Taken together, these results suggested that elevating bioactive GA levels in cotton fibers promoted secondary cell wall deposition.

### Sucrose synthase expression in response to elevated GA levels in fibers and hypocotyls

**Figure 3:**
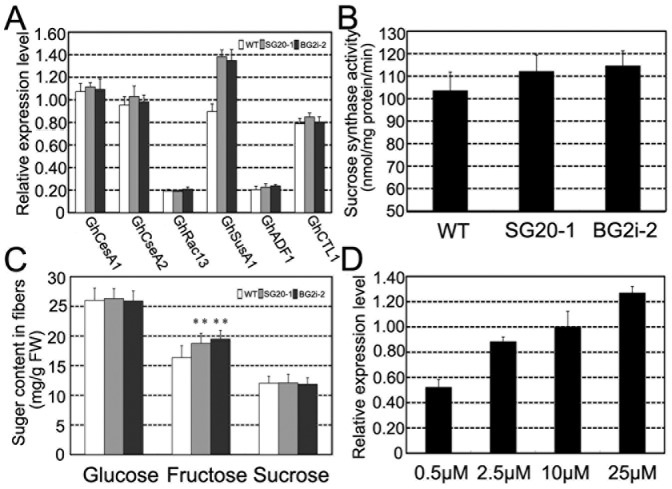
Sucrose synthase expression in GA overproduction cotton fibers. (A) Quantitative RT-PCR expression analyses of secondary cell wall related genes in 20-dpa fibers. *GhCesA1*, U58283; *GhCesA2*, U58284; *GhRac13*, S79308; *GhSusA1*, HQ702817; *GhADF1*, DQ088156 and*GhCTL1*, AY291285. (B) Sucrose synthase activity in 20-dpa cotton fibers. (C) Sugar contents in 20-dpa cotton fibers, n = 3. Double asterisks (**) represent significant differences (*t* test) at p = 0.01 compared with the wild type cotton. (D) *GhSusA1* expression levels of *in vitro* cultured fibers treated with GA_3_ of various concentrations.

To reveal the possible mechanism for GAs to control secondary cell wall deposition, we investigated transcript levels of six genes related to secondary cell wall biosynthesis, including *GhCesA1*, *GhCesA2*, *GhRac13*, *GhSusA1*, *GhADF1* and *GhCTL1* [6], [7], [35], in 20-dpa fibers. Only sucrose synthase gene (*GhSusA1*) showed significant increase in transgenic cottons (Figure 3A). The relative transcript levels of *GhSusA1* in SG20-1 and BG2i-2 fibers were 53% and 50% higher than the control, respectively. Biochemical analysis demonstrated that the sucrose synthase activities in 20-dpa fibers of SG20-1 and BG2i-2 increased 8.3% and 10.7%, respectively, compared to the control (Figure 3B). Meanwhile, the concentration of fructose, a direct product of sucrose synthase, was significantly higher in SG20-1 and BG2i-2 fibers (Figure 3C). Furthermore, we found *GhsusA1* transcript in cultured fibers was increased with GA_3_ concentrations in ovule culture media (Figure 3D). The result of fiber culture, along with the observations on the mature fibers, implied that GA may promote cellulose biosynthesis and secondary cell wall deposition through up-regulation of the expression of sucrose synthase.

**Figure 4:**
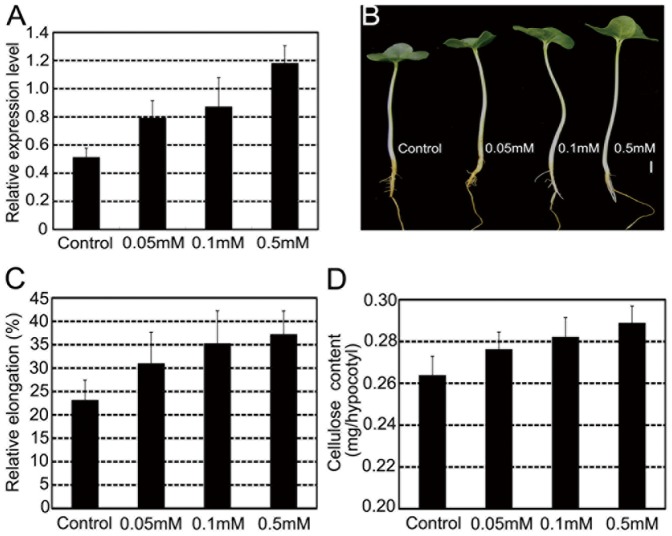
Sucrose synthase expression and cellulose biosynthesis in GA-treated hypocotyls. Seedlings wereimmersed in distilled water (pH6.0) or GA_3_ solutions (0.05 mM, 0.1 mM and 0.5 mM, pH6.0).(A) Quantitative RT-PCR analysis of *GhSusA1* transcript in hypocotyls. (B) Effects of GA_3_ treatment on cotton seedling growth. (C) Relative elongation of hypocotyls. (D) Cellulose amount in GA-treated hypocotyls.

Like cotton fibers, hypocotyls that undergo rapid cell elongation require high-speed formation of cellulose. To investigate if same response takes place in hypocotyls, we detected the expression of sucrose synthase in the GA-treated hypocotyls. *GhSusA1* transcript levels in hypocotyls were significantly enhanced along with increase of GA_3_ (Figure 4A). Meanwhile, elongation and cellulose deposition were accelerated (Figure 4B–D). These results further supported that GA promoted cellulose biosynthesis and secondary cell wall deposition through up-regulation of sucrose expression.

## Discussion

Genetically manipulating enzymes involved in GA biosynthesis and catabolism provided an effective strategy to regulate GA homeostasis in plants [15]–[20]. Our work demonstrated that in addition to up-regulation of *GA20ox*, down-regulation of *GA2ox* is another effective way to increase the active GA levels in plant tissues (Figure 1E and F). However, there may be a subtle difference between the two strategies. Up-regulation of GA20ox not only increased the GA level, but also changed the composition of some active GAs by promoting the non-13-hydroxylation pathway for producing 13-H GA_4_ rather than 13-OH GA_1_ (Figure 1E and F) [17], [19], [20]. On the contrary, in our study, down-regulation of GA2ox elevated the GA_1_ instead of GA_4_ (Figure 1E and F). The exact physiological effects of different bioactive GAs on cotton fiber development are still to be elucidated.

Cotton fibers that undergo rapid elongation and intense cellulose synthesis represent a strong sink that competes with the developing embryos and endosperms in a single ovule [1], [3], [5], [6], [8], [30], [36], [37]. It was revealed that sucrose synthase played important roles in carbon partition during fiber development. Suppression of sucrose synthase gene (*SS3*) inhibited fiber initiation and elongation [30], while over-expression of a potato sucrose synthase gene in cotton enhanced leaf expansion, early seed development and fiber elongation [5]. Recently, Jiang and coworkers cloned a novel cotton sucrose synthase gene (*GhSusA1*), which might be a key regulator of sink strength in cotton. Over-expression of *GhSusA1* significantly enhanced cell wall thickening during secondary wall formation stage, and improved fiber length and strength [6]. In this study, we revealed that enhancement of GA level in cotton fibers led to an increase of sucrose expression (*GhSusA1*) gene expression, and promoted cellulose biosynthesis and secondary cell wall deposition (Figure 2 and 3). Moreover, GA-induced *GhSusA1* up-regulation also found in cultured fibers and hypocotyls (Figure 4 and S5). GA has long been considered as an important regulator of sink strength in plants, but the molecular basis of how GA enhances the partitioning of carbon assimilates to sink tissues is still unknown [38], [39].Our data offer an experimental evidence for the relationship between GAs and sucrose synthase.

Previous studies showed that exogenous application of GAs or constitutively increased endogenous GA levels promoted fiber initiation and elongation [17], [21]. However, in this study we did not find significant improvement in fiber length of the transgenic GA-elevated fibers (TableS2). A possible explanation for this phenomenon is that the enhanced secondary cell wall deposition may fix the morphology of the fiber and, in turn, limit further elongation of the GA-enhanced fibers at the late stage of fiber elongation. Nevertheless, the finding that GA related regulation of sucrose synthase gives useful information to reveal the mechanism of cotton fiber development and to improve fiber yield and quality for cotton breeders.

## Supporting Information

Figure S1 **Alignment of cotton GA2ox proteins with homologous proteins.** The conserved amino acids are highlighted on black background, and similar amino acids are shown on gray background. The symbols (#) indicate Fe-binding sites, and the asterisks(_*_) indicateputative 2-oxoglutarate-interaction sites. GhGA2ox1-6, Cotton GA 2-oxidases (HQ891930-HQ891935, respectively); SoGA2ox1-3, Spinach GA 2-oxidases (AAN87571, AAN87572 and AAX14674, respectively); AtGA2ox1, 4 and 7, Arabidopsis GA 2-oxidases (CAB41007, AAG51528 and AAG50945, respectively).So, *Spinaciaolracea*; At, *Arabidopsis thaliana*.
(TIF)

Figure S2 **Phylogenetic relationship of GhGA2ox with other GA2ox, GA20ox and GA3ox.** GenBank accession nos. are as follows: GhGA2ox1-6, HQ891930-HQ891935, respectively; SoGA2ox1-3, AAN87571, AAN87572 and AAX14674, respectively; AtGA2ox1-4 and 6-8, CAB41007, CAB41008, CAB41009, AAG51528, AAG00891, AAG50945 and CAB79120, respectively; CmGA2ox, CAC83090; OsGA2ox3 and 5-9, AK101713, AK106859, AK107142, AK108802, AK101758 and AK059045, respectively; GhGA20ox1-3, AY603789, FJ623273 and FJ623274, respectively. So, *Spinaciaolracea*; At, *Arabidopsis thaliana*; Cm, *Cucurbita maxima*; Os, *Oryza sativa*.
(TIF)

Figure S3 **Expression pattern of** ***GhGA2ox*** **genes in cotton tissues.** The total RNA were prepared from different organs and tissues, including roots(Ro), hypocotyls (Hy), leaves (Le), petals (Pe), anthers (An), 0-dpa ovules (Ov), 6-dpa fibers (Fi).
(TIF)

FigureS4 **Field-grown plants of SG20-1, BG2i-2,** ***GhGA20ox1*** **-overexpressing (OG20) and wild-type (WT) cottons.** All the materials were transplanted to the field in parallel. The representative plants were photographed at 90d post germination. Bar = 30 cm.
(TIF)

Figure S5 **Quantitative RT-PCR analysis of fiber elongation related genes in 8-dpa fibers.** *GhACT1*, AF305723; *GhEXP1*, AF512539; *GhFLA1*, EF672627; *GhPEL*, DQ073046; *GhVIN1*, FL915120.
(TIF)

Table S1 **Primers used in Real-time PCR analyses.**
(DOC)

Table S2 **Fiber length and strength of mature fibers in two-year successive field trials.** Asterisk (_*_) and double asterisks (_**_) represent significant differences (*t* test, n = 6) at p = 0.05 and p = 0.01 compared with the wild type, respectively.
(DOC)

Table S3 **Plant height, Boll weight, seed index and lint index of transgenic and wild-type cotton in 2013.** Seed index, weight of 100 delinted seeds. Fiber index, weight of lints from 100 seeds. Data are shown as average±SD (n = 10).
(DOC)
