# Supplementary figures and images for: Gibberellin Overproduction Promotes Sucrose Synthase Expression and Secondary Cell Wall Deposition in Cotton Fibers
Source: PLoS One. 2014 May 9;9(5):e96537. doi: 10.1371/journal.pone.0096537 (PMC4015984; doi:10.1371/journal.pone.0096537)

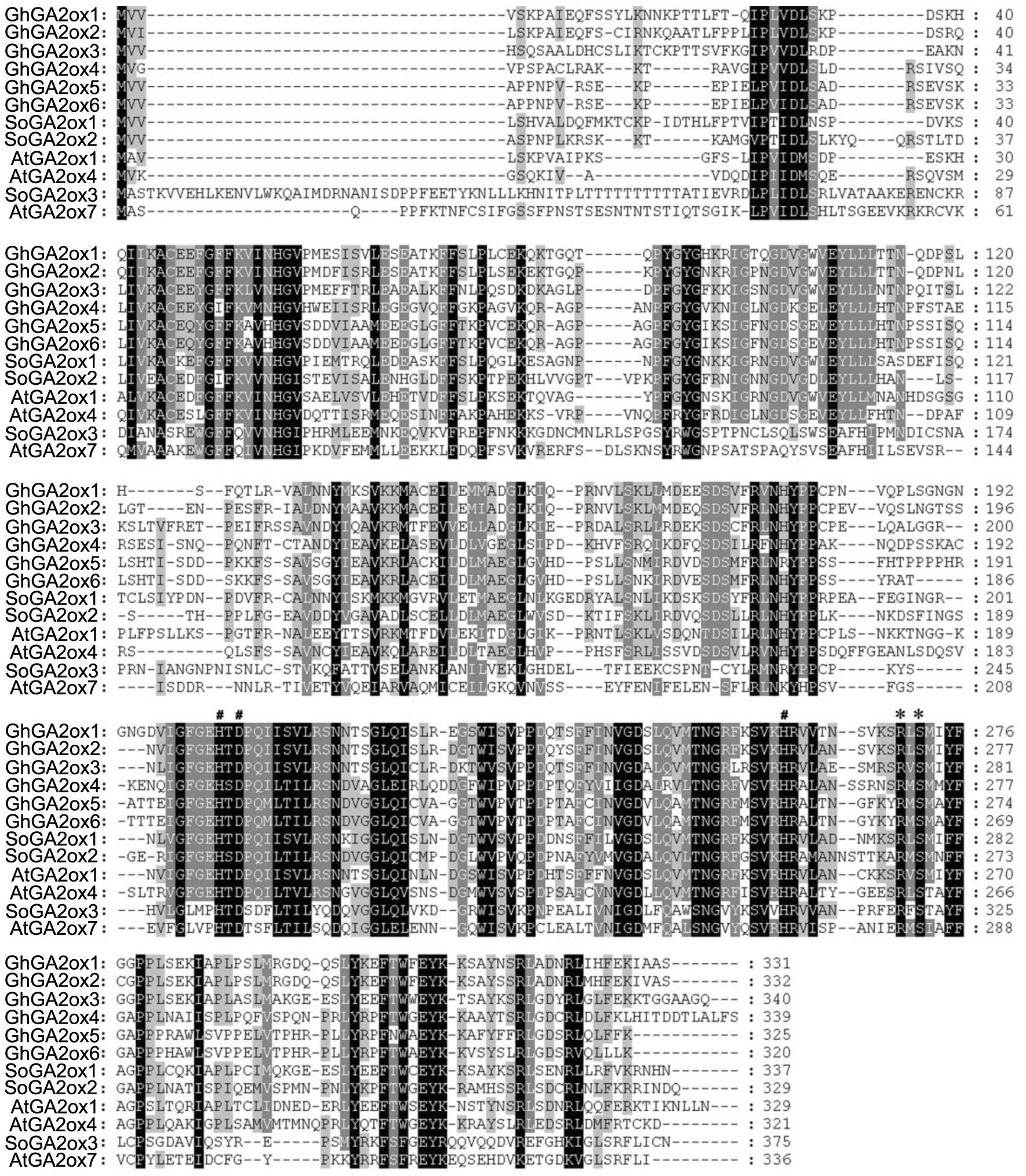

Supplement: Figure S1 — Alignment of cotton GA2ox proteins with homologous proteins. The conserved amino acids are highlighted on black background, and similar amino acids are shown on gray background. The symbols (#) indicate Fe-binding sites, and the asterisks(*) indicateputative 2-oxoglutarate-interaction sites. GhGA2ox1-6, Cotton GA 2-oxidases (HQ891930-HQ891935, respectively); SoGA2ox1-3, Spinach GA 2-oxidases (AAN87571, AAN87572 and AAX14674, respectively); AtGA2ox1, 4 and 7, Arabidopsis GA 2-oxidases (CAB41007, AAG51528 and AAG50945, respectively).So, Spinaciaolracea; At, Arabidopsis thaliana. (TIF) [file pone.0096537.s001.tif]

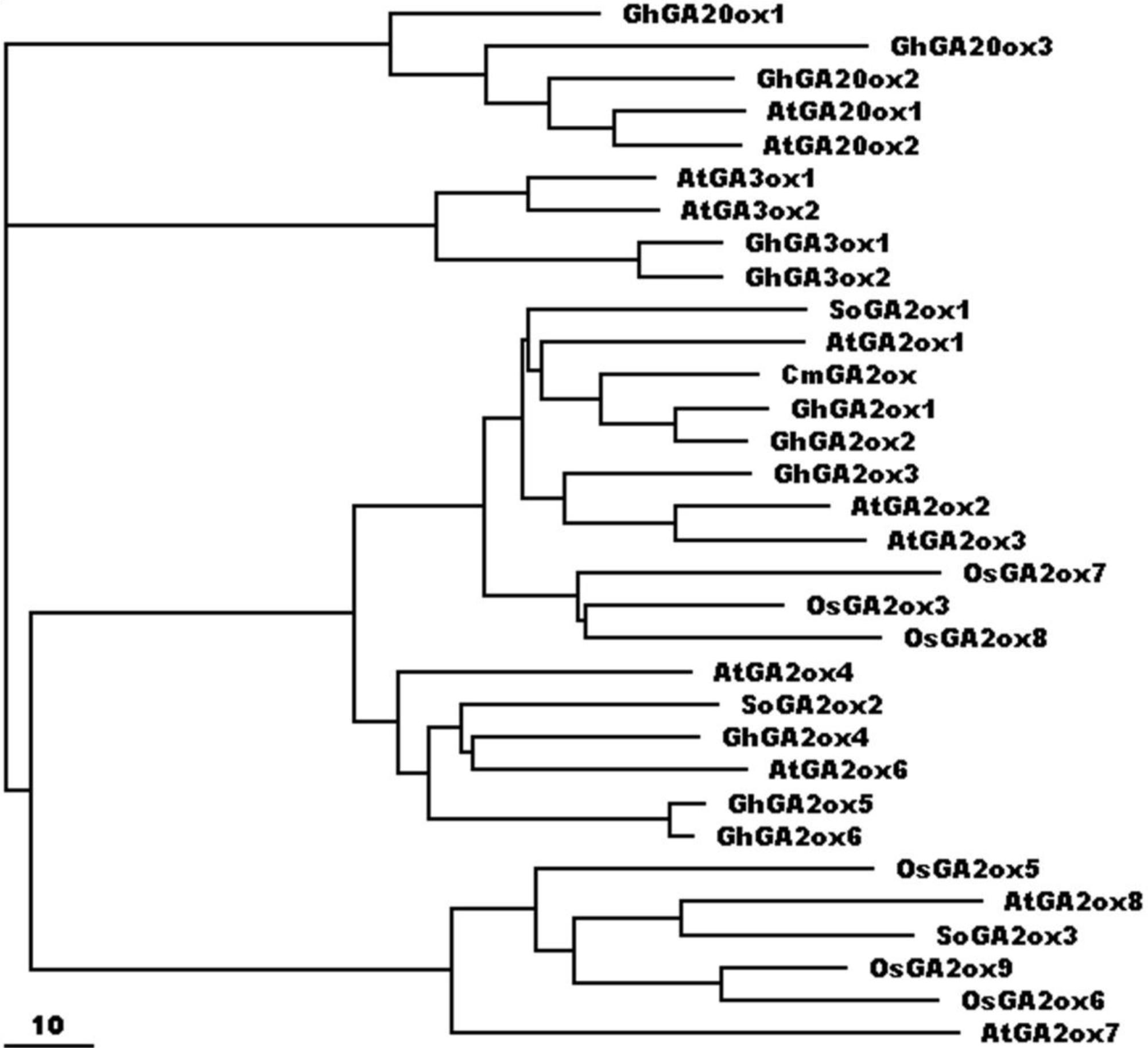

Supplement: Figure S2 — Phylogenetic relationship of GhGA2ox with other GA2ox, GA20ox and GA3ox. GenBank accession nos. are as follows: GhGA2ox1-6, HQ891930-HQ891935, respectively; SoGA2ox1-3, AAN87571, AAN87572 and AAX14674, respectively; AtGA2ox1-4 and 6-8, CAB41007, CAB41008, CAB41009, AAG51528, AAG00891, AAG50945 and CAB79120, respectively; CmGA2ox, CAC83090; OsGA2ox3 and 5-9, AK101713, AK106859, AK107142, AK108802, AK101758 and AK059045, respectively; GhGA20ox1-3, AY603789, FJ623273 and FJ623274, respectively. So, Spinaciaolracea; At, Arabidopsis thaliana; Cm, Cucurbita maxima; Os, Oryza sativa. (TIF) [file pone.0096537.s002.tif]

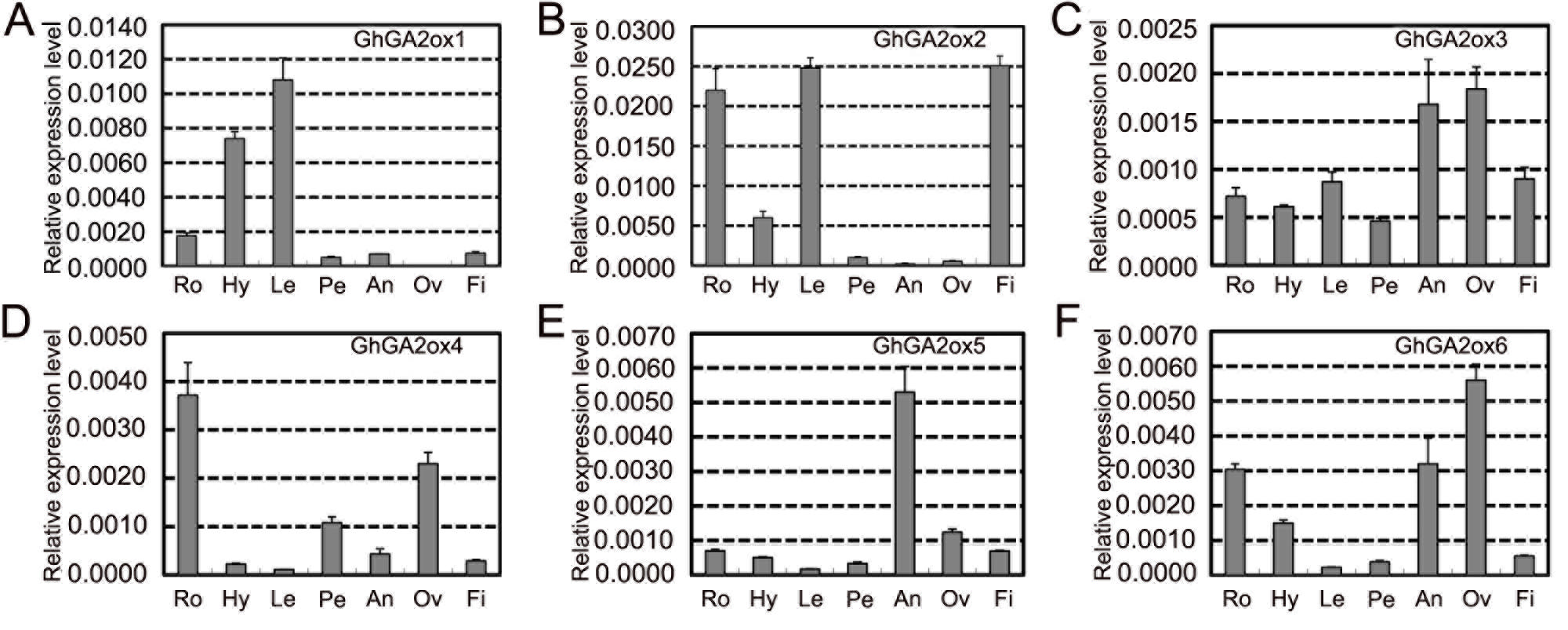

Supplement: Figure S3 — Expression pattern of GhGA2ox genes in cotton tissues. The total RNA were prepared from different organs and tissues, including roots(Ro), hypocotyls (Hy), leaves (Le), petals (Pe), anthers (An), 0-dpa ovules (Ov), 6-dpa fibers (Fi). (TIF) [file pone.0096537.s003.tif]

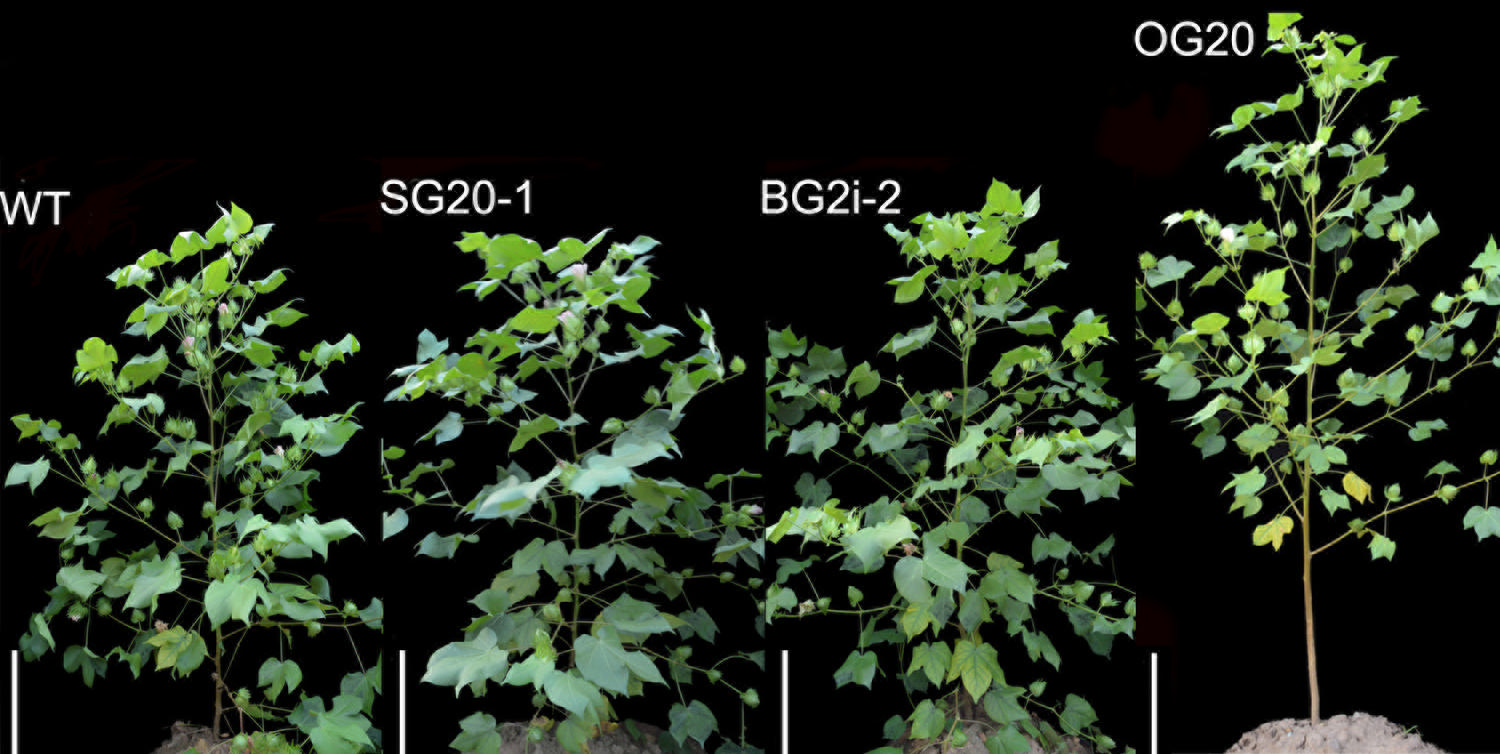

Supplement: FigureS4 — Field-grown plants of SG20-1, BG2i-2, GhGA20ox1 -overexpressing (OG20) and wild-type (WT) cottons. All the materials were transplanted to the field in parallel. The representative plants were photographed at 90d post germination. Bar = 30 cm. (TIF) [file pone.0096537.s004.tif]

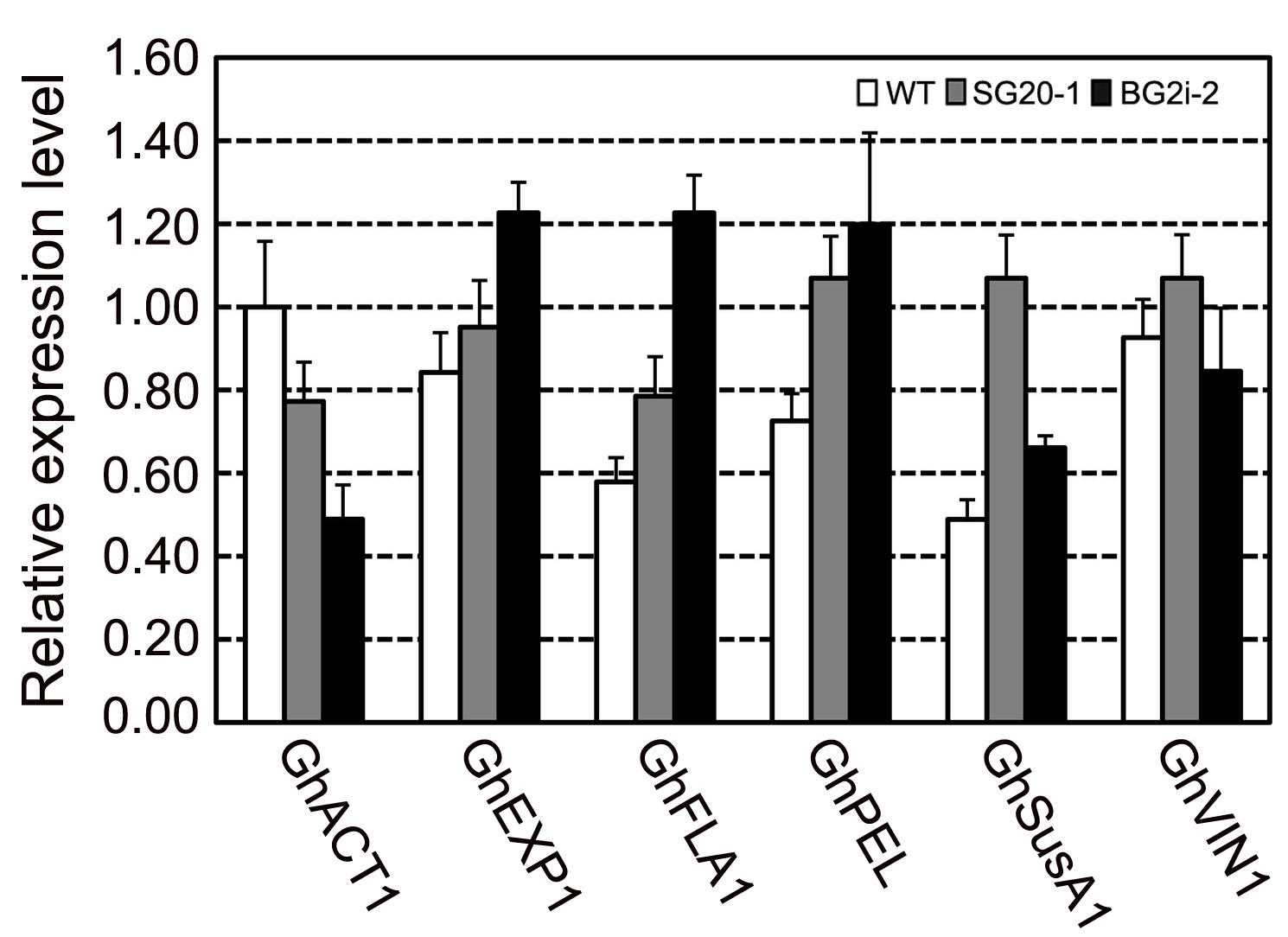

Supplement: Figure S5 — Quantitative RT-PCR analysis of fiber elongation related genes in 8-dpa fibers. GhACT1, AF305723; GhEXP1, AF512539; GhFLA1, EF672627; GhPEL, DQ073046; GhVIN1, FL915120. (TIF) [file pone.0096537.s005.tif]
