# Supplementary material for: Gibberellin Overproduction Promotes Sucrose Synthase Expression and Secondary Cell Wall Deposition in Cotton Fibers
Source: PLoS One. 2014 May 9;9(5):e96537. doi: 10.1371/journal.pone.0096537 (PMC4015984; doi:10.1371/journal.pone.0096537)
Supplement: Table S1 — Primers used in Real-time PCR analyses. (DOC) [file pone.0096537.s006.doc]

| Gene | Forward primer | Reverse primer | |
| --- | --- | --- | --- |
| *GhGA20ox1* | TTTACAAGAGCCGCTTGCAC | | ACAAAGCCCAGCATTGTCC |
| *GhGA2ox1* | GTGTAAAACATAGGGTAGTGA | | GTATGAGATTAAGAAGCAGCA |
| *GhGA2ox2* | TGTGAAGCATAGGGTACTGG | | GAAAAGCATAACGAGGAGCTA |
| *GhGA2ox3* | GCTTCATTAATGGCGAAAGG | | ACATAGCTACAACCTATGTC |
| *GhGA2ox4* | CGCAATCATATCTCCTCTCC | | CCTGGGAAAAGCAGGAAGTT |
| *GhGA2ox5* | CCTGGGAAAAGCAGGAAGTT | | GCGACTTAAACTCAAACCGA |
| *GhGA2ox6* | TTGCTGTATAGGCCATTCAC | | GAAAAGCATAACGAGGAGCTA |
| *GhCesA1* | TGGACTACCCGGTGGATAAGGT | | CTTTCTTGCAAAGTCGGCTGTT |
| *GhCesA2* | GCAGCAGACGATACAGAATTCG | | CGTTGTTGATTGCGTCTGAAAC |
| *GhRac13* | GTGAAGGCTGTTTTCGATGCT | | TCTCCTTTTGCAAGGCTTTCTC |
| *GhSusA1* | TGAGTGATCGGTCAAAGCC | | CGGTCTGGTCAGGGTGGTA |
| *GhADF1* | CGATGTCATAAGGAGCCGTGCCAAT | | GACCTTCTAACTTGATAACCAAATC |
| *GhCTL1* | CCGACCAAGAACGACACGTT | | ACCTCGCCCACAAACTTGAT |
| *GhACT1* | AATGGTGAAGGCCGGTTTTG | | TGCTTCCGTGAGTAGCACAG |
| *GhEXP1* | CCGTGACAGCCACCAACTTT | | TTCTGCTATCCGCAAGAATGC |
| *GhFLA1* | GGAGTTGCTGCTCTAGTTATTG | | AACAAGCAATGATGCAAAATCTT |
| *GhPEL1* | TGTCCTAATGCGGAGTGTGG | | ACTGAAGGTGGAATCGGACG |
| *GhVIN1* | CACGAAACGAGTGTATGGAGGCA | | CCTTCACATTCACTCTACTTGCA |
| *Histone3* | GAAGCTGCAGAGGCATACC | | CTACCACTACCATCATGGC |
