# Supplementary material for: Gibberellin Overproduction Promotes Sucrose Synthase Expression and Secondary Cell Wall Deposition in Cotton Fibers
Source: PLoS One. 2014 May 9;9(5):e96537. doi: 10.1371/journal.pone.0096537 (PMC4015984; doi:10.1371/journal.pone.0096537)
Supplement: Table S2 — Fiber length and strength of mature fibers in two-year successive field trials. Asterisk (*) and double asterisks (**) represent significant differences (t test, n = 6) at p = 0.05 and p = 0.01 compared with the wild type, respectively. (DOC) [file pone.0096537.s007.doc]

| Year | line | Length | Strength(cN/tex) |
| --- | --- | --- | --- |
|  | WT | 30.02±0.33 | 31.08±0.40 |
| 2012 | SG20-1 | 29.22±0.52^**^ | 30.02±0.44^*^ |
|  | BG2i-2 | 30.47±0.80 | 31.26±0.50 |
|  | WT | 29.66±0.62 | 30.02±1.92 |
| 2013 | SG20-1 | 29.29±0.37 | 28.15±2.03 |
|  | BG2i-2 | 29.72±0.54 | 28.82±1.95 |
