# Supplementary material for: Gibberellin Overproduction Promotes Sucrose Synthase Expression and Secondary Cell Wall Deposition in Cotton Fibers
Source: PLoS One. 2014 May 9;9(5):e96537. doi: 10.1371/journal.pone.0096537 (PMC4015984; doi:10.1371/journal.pone.0096537)
Supplement: Table S3 — Plant height, Boll weight, seed index and lint index of transgenic and wild-type cotton in 2013. Seed index, weight of 100 delinted seeds. Fiber index, weight of lints from 100 seeds. Data are shown as average±SD (n = 10). (DOC) [file pone.0096537.s008.doc]

| Line | Plant height(cm) | Boll weight(g) | Seed index(g) | Lint index(g) |
| --- | --- | --- | --- | --- |
| WT | 117.00±11.02 | 3.67±0.13 | 11.53±0.11 | 6.54±0.15 |
| SG20-1 | 118.57±10.82 | 3.91±0.06 | 11.83±0.23 | 6.68±0.05 |
| BG2i-2 | 115.73±10.37 | 3.65±0.21 | 11.93±0.09 | 6.84±0.11 |
